# The Peer Review Crisis Demands Radical Reform: Why Reviewers Should Become Paid Professional Referees

**DOI:** 10.7759/cureus.87817

**Published:** 2025-07-13

**Authors:** Enzo Emanuele, Piercarlo Minoretti

**Affiliations:** 1 Clinical Pathology, 2E Science, Robbio, ITA; 2 Occupational Health, Studio Minoretti, Oggiono, ITA

**Keywords:** peer reviewers, peer review in journals, reviewers, scientific publishing, workers’ compensation

## Abstract

The peer review system, fundamental to scientific quality control, faces a significant crisis. As journal editors, we often need to send up to 35 invitations just to secure two reviewers, confronting daily the collapse of voluntary participation. This reflects a critical imbalance: while publication pressure intensifies, willingness to evaluate diminishes, creating "literature elephantiasis", i.e., an overwhelming proliferation of papers exceeding human processing capacity. Current compensation models, relying on token recognition and database access, fail to incentivize quality engagement and may encourage ethically problematic practices like excessive self-citation. The unchecked infiltration of artificial intelligence into peer review, with minimal enforcement, further undermines system integrity. We propose transforming peer reviewers into professional referees, modeled on sports officiating. This radical solution involves formal training and certification for reviewers, equipping them to assess scientific merit, methodology, and ethics comprehensively. Like sports referees supported by assistants, scientific referees would collaborate with specialists - including statisticians, methodology experts, and reference checkers - ensuring thorough evaluation while distributing workload effectively. Funding would come from publishers or research funders, recognizing peer review as an essential, compensated component of the research lifecycle. Implementation faces challenges including publisher resistance and funding allocation, which we address through phased transition strategies. This professionalization addresses current inequities where conscientious scientists shoulder disproportionate reviewing burdens while others contribute minimally. Professional reviewers would view evaluation as valued career development rather than unwelcome obligation. Critics citing independence concerns overlook the sports analogy: referees maintain impartiality through professional standards despite league compensation. Quality scientific evaluation requires dedicated expertise, adequate training, and fair remuneration. Science deserves better than a system dependent on goodwill and guilt - it needs professional referees now.

## Editorial

The peer review system, long considered the gold standard of scientific quality control - a process where independent experts evaluate research manuscripts before publication - is in crisis [1, 2]. In our roles as journal editors for leading scientific publishers - including *Springer Nature*, *Frontiers*, and *Wiley* - we witness this collapse daily: finding volunteer reviewers has become a Sisyphean task. Recently, we may need to send as many as 35 invitations to secure just two reviewers for a single manuscript. This is not hyperbole but our lived editorial reality across hundreds of manuscripts annually. The responses - "lack of time," "not my expertise," or simply silence - paint a stark picture of a system buckling under its own weight.

This crisis reflects a fundamental imbalance in modern science: whilst researchers are eager to publish, few are willing to read and judge. The outcome is what we call "literature elephantiasis." The parallels between the peer review crisis and elephantiasis are striking: both involve pathological overgrowth that ultimately undermines normal function. In elephantiasis, lymphatic obstruction impedes proper drainage, causing tissues to swell far beyond healthy limits. Similarly, the peer review system - our scholarly filtration mechanism - cannot keep pace with the exponential surge of manuscript submissions. In both cases, unchecked expansion leads to dysfunction: a system so engorged that it can no longer fulfill its essential role. Post-publication review, often touted as a solution, remains haphazard and non-systematic, failing to provide the rigorous evaluation science demands [3]. The current compensation model for peer review is fundamentally broken. Reviewers volunteer their expertise without monetary reward, receiving instead access to databases like Scopus or ScienceDirect, or recognition through platforms like Publons. These tokens are largely perceived as inadequate, leading some reviewers to seek compensation through self-citations - an understandable if ethically problematic response that journals rightly prohibit. More troubling still is the creeping infiltration of artificial intelligence (AI) into peer review. Whilst using AI for reviews is forbidden, enforcement is virtually non-existent. As editors, we increasingly encounter reviews that bear unmistakable hallmarks of chatbot generation: excessive length (often exceeding 3,000 words when 500-1,000 would suffice), perfect formatting with no grammatical errors, extensive bullet points numbering 20 or more, generic comments that could apply to any paper in the field, and a peculiar detachment from the manuscript's specific content - such as critiquing statistical methods in purely theoretical papers or suggesting wet-lab validations for computational studies. In our editorial experience, these AI-generated reviews have become disturbingly common, appearing in roughly one of every ten reviews we receive. Who checks? Who enforces? The honour system upon which peer review depends is crumbling.

We propose a radical solution: transform peer reviewers into professional referees, modelled on sports officiating. Just as football matches require trained, paid referees to ensure fair play, scientific manuscripts deserve professional evaluation by compensated experts. This is not merely about payment - it is about fundamentally reconceptualising the reviewer's role in the scientific ecosystem. Under this model, reviewers would undergo specific training and certification, learning not just to evaluate scientific merit but to identify methodological flaws, statistical errors, and ethical concerns. Like sports referees who work with assistant referees and video assistant referees (VAR), scientific referees would collaborate with specialist assistants - including statistical reviewers, reference checkers, and methodology experts. This team approach would ensure comprehensive evaluation whilst distributing the workload more effectively.

The question of funding is critical but not insurmountable. Publishers, who profit substantially from the scientific enterprise [4], should bear primary responsibility for compensating reviewers. Alternatively, research funders could allocate specific budgets for peer review, recognising it as an essential component of the research lifecycle. Some journals might employ internal reviewers - trained professionals dedicated to maintaining standards - whilst others might maintain external review with proper compensation structures. Admittedly, implementing this system faces significant challenges. Publishers, particularly those with high profit margins (some exceeding 35-40%), may resist additional costs that could reduce shareholder returns. The logistics of transitioning millions of volunteer reviewers to a professional system would require careful orchestration. We propose a phased approach: beginning with pilot programs at flagship journals, establishing training curricula through learned societies, and gradually expanding as the model proves its worth. Initial funding could come from a small percentage of article processing charges or subscription fees - a 2-3% allocation would generate sufficient resources based on current industry revenues. Government research councils and major funders like the NIH, ERC, and Wellcome Trust could mandate professional review for funded research, creating market pressure for adoption.

This professionalisation would address the current inequity where a minority of conscientious scientists shoulder the reviewing burden whilst the majority never contribute. From our editorial databases, we observe that the same dedicated reviewers appear repeatedly while others never accept invitations - creating an unsustainable concentration of labour on the goodwill of a few. By creating clear pathways for reviewer training and career development, we could cultivate a cadre of expert evaluators who view peer review not as an unwelcome obligation but as a valued professional role. Critics might argue that paid reviewers could compromise independence, but the sports analogy holds: referees receive payment from leagues, not competing teams, thereby maintaining neutrality through professional protocols and systematic oversight. Importantly, reviewer compensation structures must ensure independence whilst recognising expertise [5]. The transformation will not be simple. It requires journals, publishers, funders, and the scientific community to acknowledge that quality control cannot remain an unfunded mandate. We must decide whether peer review is genuinely essential to science - and if so, fund it accordingly. The current system, where overworked academics squeeze reviews between teaching, research, and administrative duties, produces predictable results: delays, superficial evaluations, and increasing reliance on AI shortcuts. Professional reviewers, like professional referees, would deliver timely, thorough assessments because that would be their job, not an afterthought squeezed between other duties.

Science deserves better than a peer review system running on goodwill and guilt. The solutions are clear and tested: professionalise the role through systematic training, provide proper compensation, create formal recognition of reviewing expertise, and establish accountability structures modeled on successful sport officiating systems. Until we acknowledge this reality and act accordingly, we will continue sending those 35 invitations, hoping against hope that someone, somewhere, will say yes. The time for half-measures has passed. Science needs professional referees - trained, certified, compensated, and recognized for their specialized expertise - and it needs them now.

## References

[1] Quaia E, Crimì F, Baratella E (2022). Overcoming the crisis of the reviewing process: responsibility of a scientific journal. Tomography.

[2] Aczel B, Barwich AS, Diekman AB (2025). The present and future of peer review: ideas, interventions, and evidence. Proc Natl Acad Sci U S A.

[3] Hardwicke TE, Thibault RT, Kosie JE (2022). Post-publication critique at top-ranked journals across scientific disciplines: a cross-sectional assessment of policies and practice. R Soc Open Sci.

[4] Walter P, Mullins D (2019). From symbiont to parasite: the evolution of for-profit science publishing. Mol Biol Cell.

[5] Cheah PY, Piasecki J (2022). Should peer reviewers be paid to review academic papers?. Lancet.

